# Cardiac repair and regeneration: cell therapy, in vivo reprogramming, and the promise of extracellular vesicles

**DOI:** 10.1038/s12276-025-01549-3

**Published:** 2025-10-01

**Authors:** R. M. Imtiaz Karim Rony, Joshua D. Tompkins

**Affiliations:** Department of Diabetes Complications and Metabolism, Arthur Riggs Diabetes and Metabolism Research Institute, City of Hope, Duarte, CA USA

**Keywords:** Regeneration, Stem-cell therapies

## Abstract

Therapeutic interventions to replenish lost cardiomyocytes and recover myocardium functions following ischemic myocardial infarction (MI) remain major goals in the cardiac regeneration field. Clinical trials harnessing autologous or allogeneic cell therapy approaches from both cardiac and noncardiac cells sources, thus far, demonstrate marginal improvement. Moreover, complications such as arrythmias and graft rejections associated with cellular or organ-based therapies continue to prevail. Extracellular vesicles, on the other hand, are cell-derived, nano-sized, cargo-containing biomolecules that have emerged as potent alternatives to cell-based cardiac regeneration/replacement therapy. Recent studies demonstrate that most stem-cell-derived extracellular vesicles (Stem-EVs) are nonimmunogenic and carry cardioprotective therapeutic cargos. Moreover, administration of multiple Stem-EV types in animal models of acute MI results in reduced inflammation, apoptosis, smaller infarct size and improved cardiac functionality. With recent developments, engineered Stem-EVs with enhanced cardiac targeting, prolonged circulation and recombinant therapeutic cargos may tilt the cardiac regeneration field toward these novel cell-free biologics. Here we provide a brief overview of current approaches to repair and replenish damaged cardiomyocytes following MI via cell therapy and in vivo reprogramming, and we delve deeply into the therapeutic potentials of Stem-EVs in cardiac repair and regeneration.

## Stem extracellular vesicles transform cardiac regeneration strategies

Heart disease is a leading cause of death worldwide, with heart failure being a major concern. Current treatments help manage symptoms but do not address the root cause: the loss of heart muscle cells (cardiomyocytes). This Article reviews new approaches to regenerate heart tissue after a heart attack. Researchers are exploring cell therapy, where cells are transplanted to repair the heart, and in vivo reprogramming, which involves converting existing cells into heart cells directly in the body. Another promising area is the use of extracellular vesicles, tiny particles released by cells that can carry healing signals to damaged heart tissue. These EVs can be derived from various stem cells and have shown potential in reducing inflammation and promoting heart repair in animal studies.

This summary was initially drafted using artificial intelligence, then revised and fact-checked by the author.

## Introduction

**Fig. 1:**
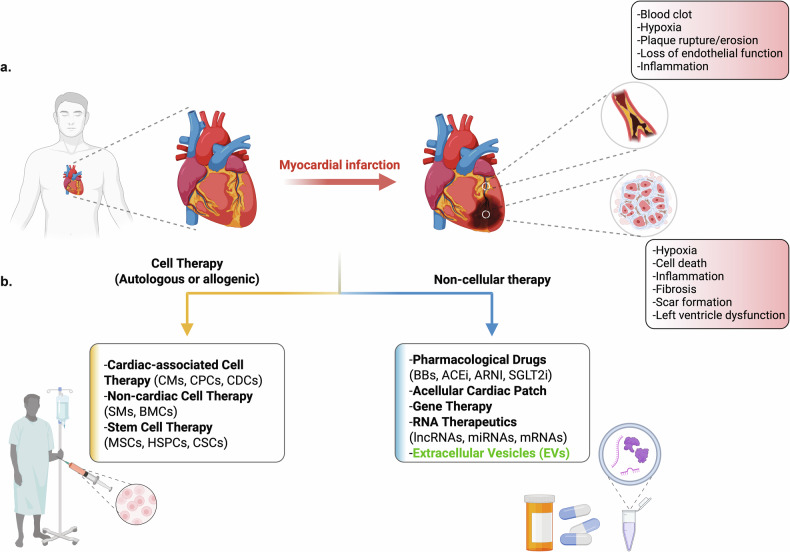
MI, loss of functional CMs, and therapeutic approaches to replenish and repair the injured heart. **a** A diagram highlighting the major pathophysiological incidents in ischemic artery and infarcted myocardium following acute ischemic MI. A significant loss of CMs combined with local inflammatory milieu, extensive fibrosis and subsequent pathological remodeling of the myocardium ultimately leads to heart failure in patients. **b** Pharmacological drugs and mechanical devices mostly assist in mitigating the pathophysiological complications associated with MI. Conversely, the injured myocardium could potentially be repaired and replenished through autologous or allogeneic cell-based therapies or noncellular approaches such as EVs or gene therapy. ACEi, angiotensin-converting enzyme inhibitors; ARNI, angiotensin receptor neprilysin inhibitor; BB, beta blockers; CSCs, cardiac stem cells; SMs, smooth muscle cells. The figure was created with BioRender.com. Rony, R. (2025).

Cardiovascular disease (CVD) consistently ranks as the leading cause of death worldwide, surpassing chronic diseases such as cancer and diabetes. Heart failure due to ischemic myocardial infarction (MI) is the primary contributor to high CVD-associated mortality (Fig. 1a). Despite the substantial healthcare expenditure incurred related to heart failure (about US$43 billion in 2020, USA alone)^1^, the post-diagnosis, 5-year survival rate for heart failure is merely ∼50% (ref. ^2^). Current pharmacological drugs, such as angiotensin-converting enzyme inhibitors, angiotensin II receptor blockers or beta blockers, mineralocorticoid receptor antagonists, angiotensin receptor neprilysin inhibitor and sodium–glucose cotransporter-2 (SGLT2) inhibitors, mostly mitigate heart failure pathophysiology/reoccurrence by reducing high blood pressure, dilating blood vessels, reducing heart rate and managing fluid buildup^3^ (Fig. 1b). Mechanical devices (for example, left ventricular assist device) relieve the asymmetric mechanobiological stress on the heart and facilitate improved blood circulation from the left ventricle^3^. Unfortunately, none of these current therapies address a primary impetus of heart failure—significant loss of cardiomyocytes (CMs), the primary contractile unit within the myocardium (Fig. 1a). Hence, the only definitive cure for end stage heart failure is a heart transplant, and this entails substantial healthcare costs and the prolonged use of immunosuppressive drugs to avoid transplant rejection.

The adult human heart is the epitome of structural and functional harmony, with its diverse cellular architecture, CMs, fibroblast cells, endothelial cells, pericycle cells and immune cells all coalesce into an organ vital to sustaining human life^4,5^. There are about 3.2 billion CMs in the heart, and these cells exhibit low turnover rates of <1% per year^6^. Considering an acute MI incident leads to the death of approximately 1 billion CMs^7^, a significant induction of CMs proliferation in vivo or the exogenous transplantation of functional CMs (Fig. 1b) are among the few solutions to replenish the infarcted heart and restore myocardium function^7^.

**Fig. 2:**
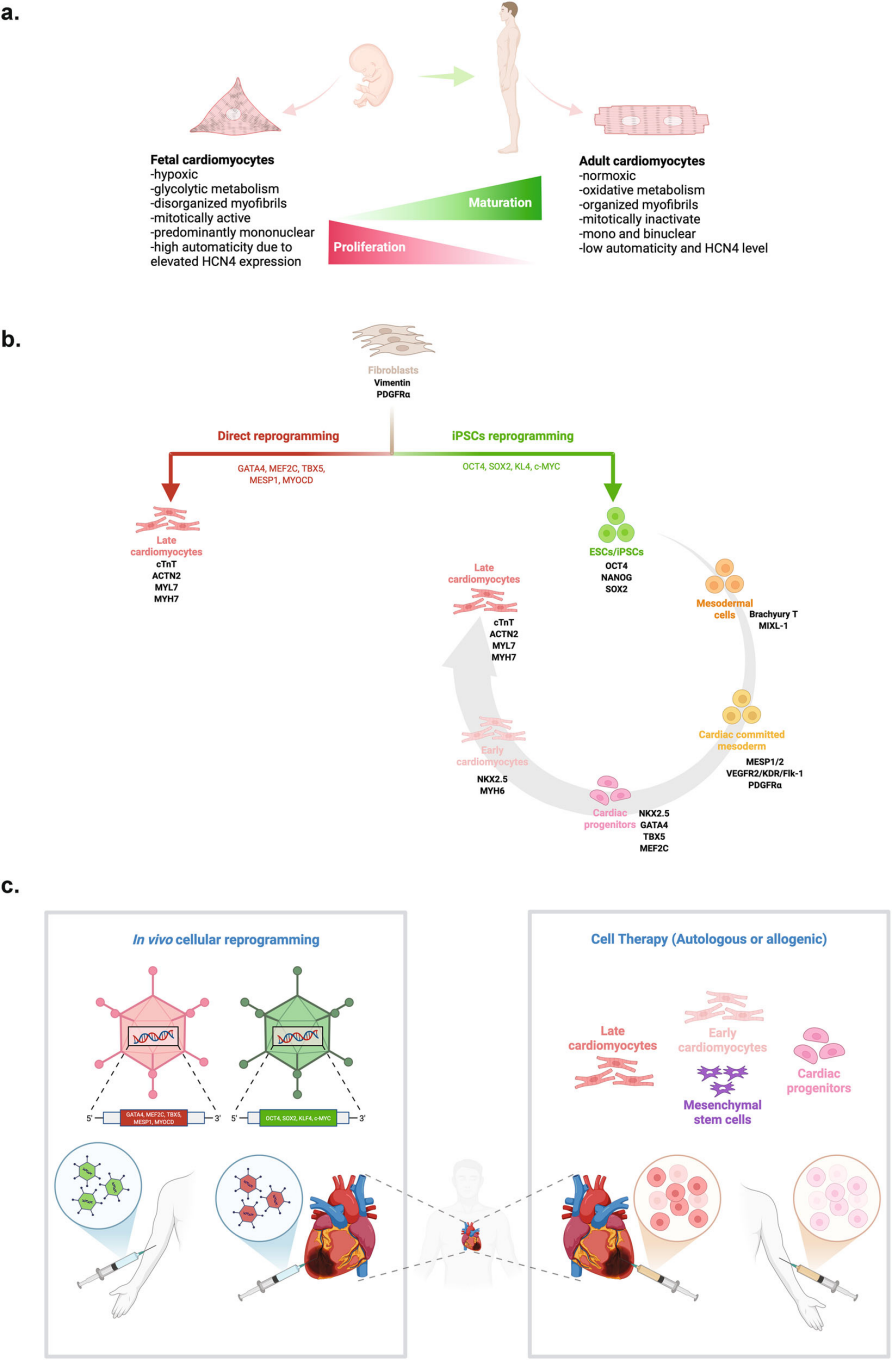
In vitro-differentiated or in vivo-reprogrammed CMs as viable sources for cardiac cell therapy. **a** A diagram comparing key differences between neonatal and adult CMs and illustrating how the neonatal-to-adult CMs maturation is associated with decreased cell division. The lack of adequate regenerative potential in adult human CMs necessitates in vitro- or in vivo-derived CMs for cardiac cell therapy. **b** A diagram illustrating key developmental stages underlying the in vitro generation of embryonic stem cells/iPSC-derived or directly reprogrammed CMs for cardiac cell therapy. In vitro-generated CMs resembles neonatal CMs and may require further maturations before in vivo transplantation. In addition, directly reprogramming resident noncardiomyocyte cells (for example, CFs) into functional CMs in vivo continues to be a major goal of cardiac regenerative medicine. **c** A diagram illustrating potential application of in vivo reprogramming of noncardiac cells into functional CMs via nonintegrating viral vectors, for example, AAV (left) or cell therapy approach employing in vitro-derived CMs or CPCs (right) in patients with MI. The figure was created with BioRender.com. Rony, R. (2025).

The first evidence of in vivo mammalian heart regeneration was reported by Porrello et al. in 2011^8^. Following cardiac injury (15% loss of the left ventricular myocardium), 1-day-old mice were able to regenerate CMs from preexisting CMs and restore the injured myocardium within 21 days^8^; however, this remarkable cardioregenerative potential is rapidly lost by day 7^8^. Inducing MI in 8-week-old mice fails to elicit resident CMs proliferation beyond baseline^9^. Between week 10 and 22 months, the CMs proliferation rate in mice continues to wane—from 5.5% to 2.6% per year, respectively^10^. In human, CMs also experience a progressive loss of their proliferation capacity during postnatal development (Fig. 2a); as a result, the typical CMs turnover rate of 1% at the age of 25 declines to 0.45% by age 75 years^11^. Collectively, these observations suggest that the naive cardiac-regeneration potential of the heart only persists for a very brief and early period of neonatal development.

With the identification of key regulators and signaling pathways implicated in the cell-cycle/proliferation arrest of CMs-such as p38 MAPK pathway, Hippo pathway, miR-15 family, MEIS1, and oxidative stress—preclinical studies targeting these regulators/mechanisms to promote CM proliferation have demonstrated notable cardioregenerative benefits (for a detailed overview of various pathways and mechanisms modulating CMs proliferation, please refer to refs. ^12–14^). However, human clinical applications for these regulators, through gene therapy (for example, knockdown or overexpression), remains limited in scope and will necessitate substantial development, especially given that no cardiac gene therapy product has been approved so far^15^.

Cell therapy approaches to repopulate infarcted myocardium and restore its function are clinically feasible (Fig. 2c, right). First generation cardiac cell therapy, which includes preclinical and clinical trials, investigated the autologous/allogeneic transplantation of noncardiac cells (for example, skeletal myoblasts and endothelial cells) and cardiac-associated cell types (for example, cardiac fibroblast, cardiac precursor/progenitor cells (CPCs)) and cardiosphere-derived cells (CDCs))^16^ (Fig. 1b). Despite strong in vitro and preclinical results, most clinical trials so far have failed to improve clinical outcomes; patients often exhibited marginal to no change in left ventricular ejection fraction (LVEF) and frequently developed cardiac arrythmias^16^. Human mesenchymal stem cells (MSCs), distinguished for their proangiogenic, anti-inflammatory and cardiogenic-differentiation potential, have also been extensively investigated for cardiac repair^17^. Some MSC-based clinical trials are still ongoing but results from POSEIDON or PROMETHEUS clinical trials have already demonstrated improved cardiac functionality and a lack of arrythmia in treated patients^16^. Since many MSC studies demonstrate poor long-term survival of transplanted cells^16^, MSC-mediated cardioprotective benefits probably originate from paracrine signaling from transplanted MSCs to CMs and the hosts immune system^18^.

The advent of human embryonic stem cells (hESCs) and patient-specific human induced pluripotent stem cells (hiPSCs) revolutionized the landscape of regenerative medicine^19,20^. With improved cardiac differentiation protocols, hESC/hiPSC-derived CMs (hPSC-CMs) were initially regarded as the best remedy to repair the infarcted heart^19,20^ (Fig. 2b, c). Though high hopes remain, many studies underscore that in vitro-differentiated hiPSC-CMs resemble neonatal CMs, both structurally and functionally (Fig. 2a) (for further details on the structural and functional differences between neonatal and adult CMs and strategies for in vitro maturation please refer to refs. ^21–23^). As such, in vivo transplantation of hPSC-CMs in murine MI model and/or human patient often leads to arrythmias^24,25^, and poor in vivo retention^26,27^.

To avoid CMs transplantation complications, direct reprogramming of noncardiac cells (for example, cardiac fibroblast (CF)) into induced cardiomyocyte-like cells (iCMs) has also been explored (Fig. 2b). Unlike hPSC-CMs, iCMs bypass the pluripotent state and are directly transdifferentiated via forced expression of cardiac-specific transcription factors including GMT (GATA4, Mef2C and Tbx5) or GHMT (GATA4, Hand2, Mef2C and Tbx5)^28^. Moreover, iCMs exhibit molecular phenotypes that resemble adult CMs^29^. Considering most adult somatic cell types are refractory to cellular reprogramming (<10% reprogramming efficiency), in vivo reprogramming of CFs in patients with MI remains promising but far from routine clinical use (Fig. 2c, left). Future advancements on tissue- and cell-specific targeted delivery of reprogramming factors combined with the development of more potent reprogramming techniques/factors should clinically translate cellular reprogramming based cardiac therapy for human clinical applications.

Extracellular vesicles (EVs) represent a group of membrane-enclosed, nano-sized, cell-secreted particles that have evolved as natural mediators of cell–cell communications, and these have been heavily implicated in organismal development and physiology^30^. EVs are broadly categorized into three major types based on their size and biogenesis pathway–exosomes (50–150 nm), microvesicles (150–1000 nm) and apoptotic bodies (1000–5000 nm)^30^. Exosomes originates from the fusion of multivesicular bodies to the plasma membrane whereas the outward budding of plasma membrane, tethered with cargo components, generates microvesicles^30^ (for a detailed overview of EVs and their biogenesis, please refer to references^30,31^). Recently, minimal information for studies of EVs (MISEV2023) guidelines by the International Society for Extracellular Vesicles (ISEV) have suggested referring EVs based on their sizes to avoid biogenesis associated EV nomenclature: small EVs (sEVs) are 50–150 nm in diameter, which predominately include exosomes whereas large EVs are defined as particles >200 nm^32^. As most studies still conform to biogenesis nomenclature of EVs, we will continue to refer to exosomes in this study, and when referring to EVs here forward, we are referring to sEVs, a population known to contain predominately EVs of endosomal origin, unless explicitly defined otherwise.

**Fig. 3:**
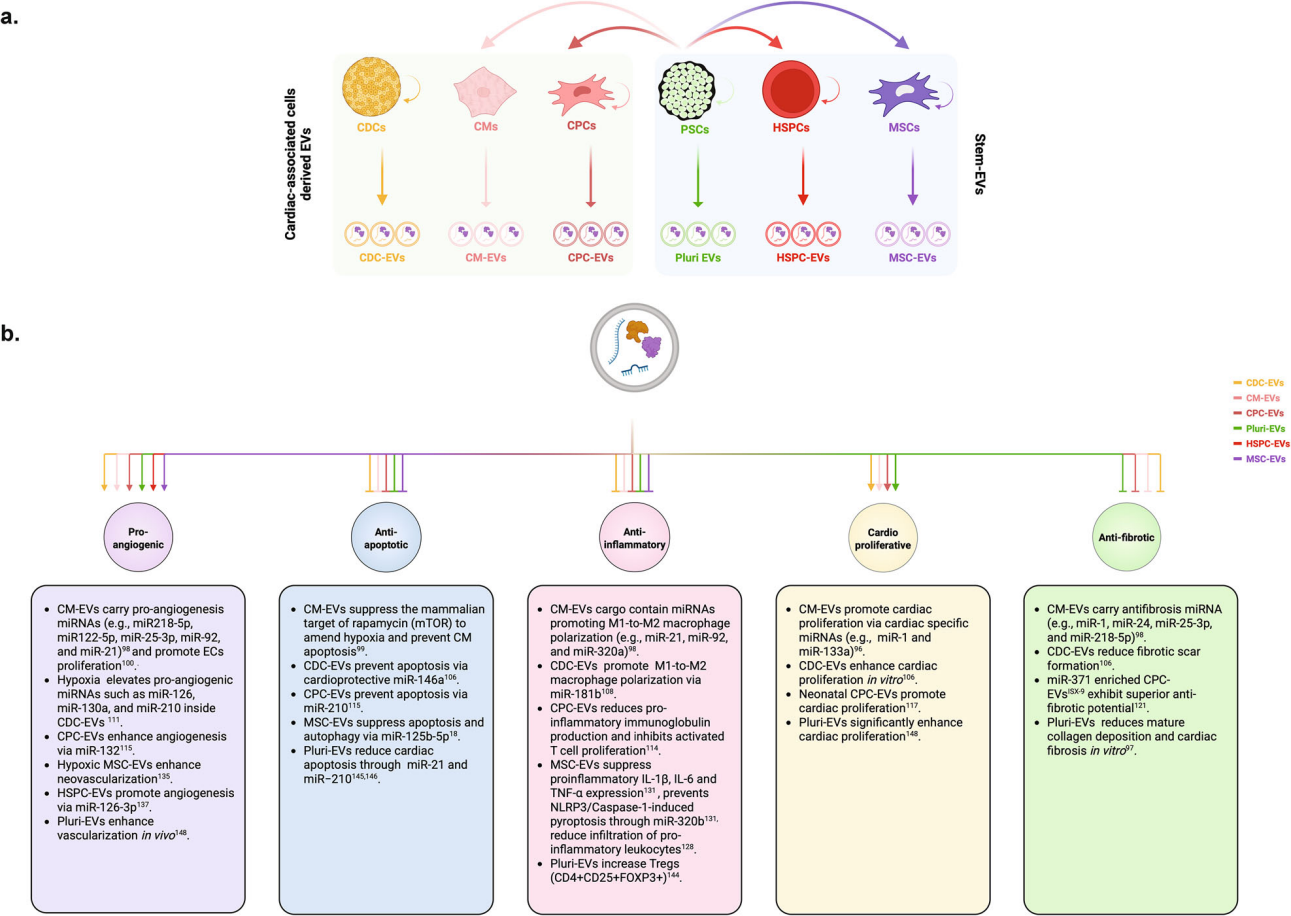
Cardiac-associated cells and Stem-EVs for cardiac repair and generation. **a** A diagram illustrating different sources of stem cells, stem-cell-derived CPCs or CMs and their EVs with demonstrated cardioprotection in preclinical animal models. EV cargos reflect the biomolecular reservoir (miRNAs, mRNAs, proteins, metabolites and ligands) of the parent cells. **b** A diagram highlighting the major cardioprotective effect reported for Stem-EVs and their derivatives. Therapeutic EVs, such as Stem-EVs, exert pleiotropic cardioprotective effects through multiple mechanism, mediated by diverse cargo components such as miRNA, factors and metabolites. Cardioprotective benefits between Stem-EVs and their parental cells are often comparable. PSCs, pluripotent stem cells. The figure was created in BioRender.com. Rony, R. (2025).

So far, EVs have been isolated from virtually all cell types and biological fluids^33^. Mounting evidence suggest that EVs not only participate in paracrine signaling in vivo but they also carry cargoes (miRNAs - micro RNAs, mRNAs, lncRNAs - long non-coding RNAs, proteins, metabolites, ligands and so on) which benefit recipient cells^33^. In this regard, EVs derived from different stem cell sources (Stem-cell-derived extracellular vesicles, Stem-EVs) (Fig. 3a) have attracted much attention for their anti-inflammatory, antiapoptotic and angiogenic benefits, where EV administration often mimics the paracrine effects of their parental cells^34^ (Fig. 3b). Several studies affirm that EVs are also nonimmunogenic and the administration of EVs from multiple stem cell sources in animal models of acute MI show reduced inflammation, apoptosis, infarct size and improved cardiac functionality (Fig. 3b).

**Fig. 4:**
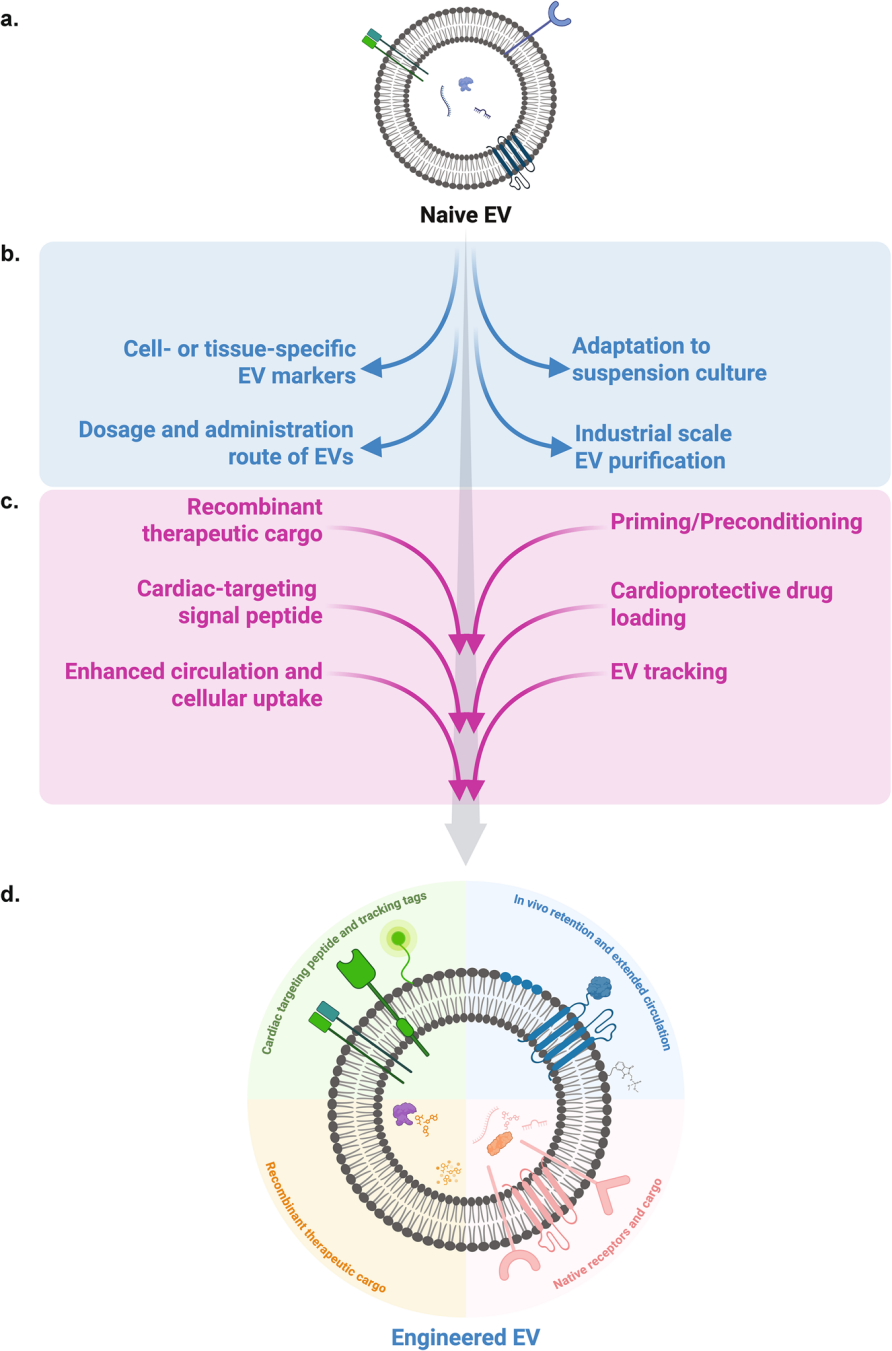
Engineered EVs as next-generation biologics for cardiac therapy. **a** Most therapeutic EVs such as Stem-EVs contain native cardioprotective cargo; however, further engineering of the parent cells could bolster Stem-EVs therapeutic efficacy. **b** A diagram highlighting limitations to Stem-EVs for clinical applicability. **c** The diagram illustrates potential approaches to augment native Stem-EVs for greater therapeutic efficacy in cardiac therapy. **d** Top left: parental stem cell lines can be engineered to incorporate cardiac-targeting peptide and tracking tags for targeted EV delivery and post-administrative tracking. Bottom left: the EV-producer line can also be primed or preconditioned to enrich cardioprotective therapeutic cargoes (miRNAs, lncRNAs or proteins) and loaded with cardiogenic or anti-inflammatory drug, targeting multiple candidates. Top right: in addition, the EV membrane surface could be modified to expressed recombinant tetraspanins, as well as specialized phospholipids to maximize circulatory duration and increase in vivo retention. The figure was created in BioRender.com. Rony, R. (2025).

In this review, we summarize current therapeutic approaches to replenish lost CMs and restore myocardial function following MI. First, we will briefly review and evaluate cell therapy and in vivo cellular reprogramming approaches to replenish lost CMs and generate iCMs in vivo, respectively. Next, we focus on noncellular therapeutic approaches, particularly the role of cardiac-associated cell-derived EVs and Stem-EVs in cardiac repair and regeneration. Furthermore, we discuss in detail the current limitations within the EV field (Fig. 4b) and highlight potential future directions that may facilitate the clinical translatability of EV therapy (Fig. 4c). Overall, our discussion provides a better understanding of the therapeutic approaches needed for repairing and regenerating CMs and how Stem-EVs and cardiac lineage-derived EVs may play pivotal roles in MI therapy.

## MI at a glance

Among CVDs, MI accounts for majority of the heart failure, hospitalization and sudden cardiac death incidents^35^. At its core, MI is an irreversible pathological manifestation of coronary ischemia that typically stems from occlusion or obstruction of blood flow into the myocardium^36^ (Fig. 1a). Overall, the systematic accumulation of low-density lipoprotein and triglyceride-rich lipoproteins on the intimal layer of the blood vessel leads to oxidation of these internalized lipids and localized inflammation, which activates the endothelial cell layer, leading to infiltration of proinflammatory cells. This triggers cycles of inflammation and lipids accumulation which give rise to the formation of atherosclerotic plaques^37,38^. Eventually, the erosion of endothelial cell layer or rupture of the plaques, frequently within the coronary artery leading to thrombus (blood clot) formation, and ultimately MI^39^ (Fig. 1a).

CMs rely on atrial blood oxygen and spend 75% of their energy (ATP) for contractile functionality; thus, MI associated hypoxic shock forces them into anaerobic glycolysis, which barely meets their minimal ATP requirement^40^. Within the first hour of MI, CMs undergo numerous ultrastructural changes of cellular deterioration marked by glycogen depletion, swelled mitochondria, disintegrated sarcolemma and relaxed myofibrils^40^. Prolonged continuation of cellular deterioration under hypoxia and elevated reactive oxygen species levels in vivo triggers a combination of apoptotic, necrotic, and necroptosis pathways which ultimately culminates in substantial CMs cell death at the infarct site^41,42^ (Fig. 1a). Approximately 1 billion CMs die following an acute MI event^7^, and to compensate, the heart progressively remodels. Surviving patients will generally go on to develop heart failure^43^.

At the cellular level, MI repair follows three highly complex yet orchestrated stages: (1) the inflammatory phase, (2) the proliferative phase and (3) the repair phase^44^. In the inflammatory phase, dead CMs and damaged extracellular matrix generate a large pool of damage signals in the extracellular space and in the circulation (for example, HMGB-1, HSPs, mtDNA, cardiac myosin, HA and S100s), which are recognized as damage-associated molecular patterns (DAMPs). DAMPs are recognized by pattern recognition receptors, typically located in the infiltrating leukocytes, which trigger a cascade of inflammatory signaling at the infarct site during the first 72 h and elicit immune responses^44,45^.

In the infarcted myocardium, there is an infiltration of CD4^+^ T cells into the left ventricle, which is associated with heart failure in both human and mice^46^. To this end, regulatory T cells (T_reg_), a unique subclass of CD4^+^ T cells expressing CD25 and transcription factor FOXP3 (CD4^+^CD25^+^FOXP3^+^), have gained immense attention for their role in containing inflammation and autoimmune responses in diverse contexts^47,48^. For example, during MI, Foxp3^+^ T_reg_ cells have been found to infiltrate myocardium, protect CMs against cell death, and promote wound healing^49–51^. Thus, promising MI/heart failure interventions must involve both immune regulatory and CM regenerative effects.

In the proliferative phase, post-MI inflammatory responses are suppressed through the clearance of DAMP signals and short-lived inflammatory cells (for example, neutrophils), which promotes the activation of anti-inflammatory signals and profibrotic and angiogenic cytokines/factors by the macrophages. The elevated proliferation of CFs at the infarct site and a subset of fibroblasts transdifferentiating into myofibroblast-like cells expressing contractile proteins (for example, α-smooth muscle actin (α-SMA)), are hallmarks of the proliferative stage of MI repair^44,45^. The reparative stage commences with the deposition of structural matrix components and scar formation through fibrosis by the activated fibroblast/myofibroblast population. Ultimately, this process culminates with the maturation phase where fibroblasts are deactivated to maintain the fibrotic scar and suppress the proangiogenic signaling activated earlier^37^. For each of these stages, several mechanistic details remain under investigation.

## Therapeutic approaches to repair and regenerate the infarcted heart

From therapeutic point of view, we propose that MI incident can be conceptually stratified into four point-of-care stages for targeted therapeutic intervention: (1) early preventative stage—prevention of atherosclerosis formation and/or MI, (2) suppression of post-MI inflammation, (3) neovascularization—amending hypoxia and (4) cardiac replacement therapy—structural and functional recovery of the infarcted heart. Given that most MI incidents are usually sudden and unanticipated, post-MI therapy remains a major focus for therapeutic intervention and will be the focus of this study. Considering an acute MI incident leads to the loss of approximately one third of the resident CMs^7^, a critical driver of heart failure following MI, a major goal of cardiac regeneration field is to minimize CMs apoptosis, replenish the lost CMs and repair the infarcted myocardium (mitigate local inflammation and fibrosis and improve LVEF) as a means to recover myocardium function. So far, three major strategies have been vigorously pursued to replenish CMs and restore heart function post-MI: (1) cell therapy (Fig. 2c, right), (2) in vivo cellular reprogramming (Fig. 2b, c, left) and (3) EV therapy (Fig. 3). In the following sections, we discuss recent development across these approaches, delineating their respective advantages and limitations (Table 1).

**Table 1:** Comparative advantages and limitations of cell therapy, in vivo reprogramming, and EV-based therapy for MI.

	Cell therapy	In vivo reprogramming	EV therapy
Source of therapeutic agents	Whole cells (in vitro-differentiated or surgically isolated)	Somatic noncardiac cells reprogrammed in situ into CMs	Nanoscale particles secreted by cultured cells
Mode of administration	Surgical transplantation (cell sheet or patch) or intramyocardial injection	Intramyocardial injection of viral or nonviral reprogramming vectors	Intravenous bolus injection or intramyocardial delivery
Requirement for cellular origin	Primarily autologous	Not applicable	Autologous or allogeneic
Immunogenicity	Present, even with autologous grafts	Strong immune response due to vector delivery	Minimal to none, compared with AAVs or LNPs
Washout/clearance	High (up to ~80% of cells lost post transplantation)	Not applicable	Moderate to High (nonspecific accumulation in liver, spleen, lungs and gastrointestinal tract)
Tumorigenic potential	Low but risk remains with undifferentiated cells, for example, MSCs	Low to moderate (potentially due to uncontrolled reprogramming)	None reported
Graft or vector rejection	High (limited long-term engraftment)	Reprogramming vectors may be resisted or rejected	Not applicable
Availability/logistics	Moderate (cells can be cryopreserved and prepared in advance)	Low (requires reprogramming over weeks to months in vivo)	High (EVs can be stored at −80 °C and administered on demand)
Cost considerations	High (due to cell production and surgical requirements)	Undefined/experimental	Moderate to high (EVs could be isolated from conditioned medium from cell therapy, lowering manufacturing and storage costs)
Therapeutic efficacy	Moderate (limited by cell retention and survival)	Low to moderate (reprogramming efficiency <5–10%)	Moderate to high (dependent on EV dose, composition and delivery frequency)
Time to exert therapeutic effect	Delayed (typically weeks)	Delayed (weeks to months)	Rapid (immediate to <1 week)

### Cell therapy for cardiac repair and regeneration

The limited regenerative potentials of adult CMs combined with the drastic loss of CMs in the infarcted myocardium has spurred the development of both autologous and allogeneic cell therapy approaches aimed at replenishing the infarcted heart (Fig. 1b). Many reviews have reported recent development of cardiac cell therapy field, and these are briefly summarized next (review refs. ^16,52,53^ for additional details on cardiac cell therapy field).

One of the earliest cell therapy initiatives started in 2000 with autologous noncardiac skeletal myoblasts transplanted into the infarcted myocardium of a patient with severe ischemic heart failure^54^. The patient’s LVEF increased ~9% with no post-transplantation arrhythmia, as reported for a 5-month followed up^54^. Based on the promise of this pilot study, the myoblast autologous grafting in ischemic cardiomyopathy (MAGIC) phase I clinical trial was initiated. MAGIC was a randomized, placebo-controlled, double-blind study designed for 120 patents with heart failure. Unfortunately, the LVEF parameter, which was the primary end point, did not improve at either low- and high-skeletal myoblast dose compared with the placebo. Increased incidence of cardiac arrythmia in patients at 6-month follow up were also reported^55^.

The next noncardiac cell type tested in patients with heart failure was generic (unselected) bone marrow cells (BMCs); unfortunately, the clinical trial found no significant difference in LVEF between control and BMC-treated patients^56^. In spite of the disappointing outcome, the FOCUS-CCTRN trial^57^ and the TAC-HFT randomized trial^58^ continued to examine BMCs-based cell therapy for ischemic cardiomyopathy. However, these also ended with suboptimal results.

As opposed to generic BMCs, early clinical trials employing multipotent bone-marrow-derived MSCs (BMMSCs) achieved improved LVEF parameters in patients with MI^59,60^, which ignited a surge of interest in harnessing BMMSCs for cardiac therapy. This is evidenced by the sheer number of clinical trials currently utilizing MSCs^16^. Indeed, a meta-analysis of BMMSCs based cell therapy clinical trials (38 randomized controlled trials involving 1907 participants) suggests that BMMSCs improves LVEF and patients’ mortality without incurring transplantation associated complications^61^.

The second type of multipotent stem cells which entered cardiac cell therapy pipeline was CD133^+^ hematopoietic stem and progenitor cells (HSPCs)^62^. Intramyocardial injection of HSPCs in patients receiving coronary artery bypass grafting showed significant increase in LVEF compared with patients who underwent coronary artery bypass grafting at 6-months postoperation^62^. These promising results ultimately led to the double-blinded, randomized, placebo-controlled Cardio133 trial. This trial included 60 patients with chronic ischemic heart disease (LVEF <35%). Unfortunately, aside from minor localized improvement of infarct scar, no gross improvement on LVEF was observed in HSPC-treated patients^63^. It is possible that graft rejection and inadequate cellular paracrine activity of the transplanted HSPCs contributed to the suboptimal results.

Based on premises that cardiac-associated cells are innately programmed to assist CMs function and support overall cardiac functionality, considerable efforts were additionally devoted to establishing cardiac cell therapy. Early cardiac cell therapy trials mostly focused on harnessing ‘c-KIT^+^ cardiac stem cells’ as renewable source of CMs for heart failure remedy, either alone or combined with MSCs^16^. There is, however, controversy over the existence of cardiac resident c-KIT^+^ cardiac stem cells in vivo, and many notable trials, including TAC­HFT­II, failed to significantly improve LVEF, and/or other key endpoints^16^. Thus, attention was drawn to exploiting in vitro-generated CPCs or CMs from human pluripotent stem cell sources (hESCs/hiPSCs).

Using hESC-derived CPCs (CD15^+^, Is1^+^) embedded within a fibrin scaffold, Menasché et al. led the first-in-human clinical trial of hESCs-CPCs on six patients with severely impaired left ventricular (LV) function. Although no patients developed arrythmias or tumors, three patients displayed autoantigens and only one patient showed significant improvement in heart functionality^64^. Meanwhile, hPSC-CMs, in numerous preclinical studies, augment LVEF parameters and decrease infarct size in MI models^65^. Concerns, however, remain for CMs subtype heterogeneity and inadequate electrical coupling between immature/neonatal-like hPSC-CMs and resident adult CMs and the potential to develop arrythmias^14,66^ (Fig. 2a). In vitro CMs maturation strategies, such as longer culture time (3-4 months)^67,68^, changing culture substrate stiffness^69,70^, switching to low-glucose plus high-fatty-acid media^71,72^, electrical^73,74^ and mechanical stimulation^75,76^ may overcome some of these issues^21,77^. Further, it has been reported that overtime, transplanted hPSC-CMs acclimate to their aged environment and mature in vivo^78^. Given that studies have reported >80% initial CM transplant washout rates, retaining a substantial fraction of transplanted cells in vivo over an extended period must be achieved to maximize the therapeutic efficacy of cardiac cell therapy^26^. To facilitate, recent advancements in three-dimensional cardiac tissue engineering, in particular, the application of biomimetic three-dimensional scaffolds, which incorporate neovascularization capacity, combined with three-dimensional bioprinting of hPSC-CMs should provide a physical niche to facilitate CMs organization, retention, maturation and survival following in vivo transplantation^79^.

### In vivo cellular reprogramming for cardiac repair and regeneration

Reprogramming adult somatic cells into desired cell types represents one of the groundbreaking advancements in the field of regenerative medicine^80^. Significant efforts have also been devoted since to establishing methods to reprogram resident cardiac and noncardiac cells into functional CMs in vivo^81^. Such approaches are expected to minimize graft rejection, autoantibodies and cardiac arrythmia associated with CM transplantation. Two types of approaches have been used; reprogramming with pluripotency-associated factors into i4F^Heart^ CMs and direct reprogramming of adult somatic cells, such as CFs, into functional induced CMs (iCMs) via transdifferentiation using cardiac master transcription regulators (Fig. 2b). These strategies are discussed next.

#### Reprogramming with pluripotency factors

In 2016, a remarkable study from Izpisua Belmonte’s group demonstrated that aside from generating iPSCs from somatic cells, short-term, cyclic induction of pluripotency-associated factors Oct4, Sox2, Klf4 and c-Myc (OSKM), termed partial reprogramming, remarkably ameliorated aging-associated cellular phenotypes. Improvements to several major organs and significantly longer lifespans were observed in a premature aging mouse model (LAKI)^82^. Notably, pulsing LAKI mice with OSKM significantly enhanced the viability of vascular smooth muscles cells and reduced the development of bradycardia, which are common in LAKI mice^82^.

Subsequently, heart-specific benefits to OSKM induction were explored (i4F^Heart^). Remarkably, OSKM induction in CMs promotes both mononucleated and binucleated CMs to reenter the cell cycle and complete cell division^83^. Gene expression analysis shows i4F^Heart^ CMs resemble neonatal and embryonic day 14.5 CMs. Furthermore, transient (6 days) overexpression of OSKM before MI, 1 day after MI or even 6 days after MI markedly reduces the infarct size and significantly increases the number of EdU^+^ CMs near the infarct size. The corresponding improved LVEF suggests functional myocardium recovery^83^. However, the long-term induction of OSKM could lead to the formation of neoplasms in heart, and direct translation to human therapies will require additional development. Novel CM or CF targeting strategies which activate a controlled level of OSKM signaling in vivo are specifically needed.

#### Reprogramming with cardiac factors (iCMs)

Contrary to OSKM-based reprogramming which pushes cells toward a transient pluripotent state, direct reprogramming of adult somatic cells into other cell types, also known as transdifferentiation, is also of significant interest in cardiac regenerative medicine^81^. In the heart, CFs account for 27–50% of ventricular cell population and contribute to fibrotic scar formation and ventricular remodeling following MI^84^; hence, directly reprogramming a proportion of CFs to iCMs represents a promising approach to replenishing infarcted heart from within.

The first direct cardiac reprogramming was reported by Srivastava group in which forced expression of cardiac master transcription regulators GMT in mouse postnatal cardiac or dermal fibroblasts transdifferentiates these cells into iCMs without going through cardiac progenitor state^85^ (Fig. 2b). Resultant iCMs express the CM marker cardiac troponin T (cTnT), and their global gene expression pattern resembles postnatal CMs, which suggest that in vivo transdifferentiation of noncardiac cells into iCMs could potentially be translated into MI treatments^85^. Interestingly, the addition of Hand2 to the GMT reprogramming cocktail (GHMT—Gata4, Hand2, Mef2c and Tbx5) augments reprogramming efficiency of mouse tail tip fibroblast from 2.5% to 9.2% and CFs from 1.4% to 6.8% (ref. ^86^). Transfecting the infarcted myocardium of mice with GHMT viral constructs not only reprogrammed ~6.5% CFs into iCMs in vivo, but the mice showed significant improvement in LVEF and reduction in fibrosis and infarct size^86^. Collectively, these results indicate that in vivo reprogramming of resident CFs to repair infarcted myocardium holds great promise and remains a key research area for regenerating the damaged heart.

In humans, direct reprogramming of human fibroblast into iCMs requires GMT and two additional factors: ESRRG and MESP1^87^ (GMTEM). Overexpression of GMTEM in hESC-derived fibroblasts results in ~10% iCM cells expressing CM markers αMHC and cTNT^87^. However, GMTEM-based reprogramming of primary human dermal fibroblasts (DFs) and CFs is inefficient^87^. GMTMM (GMT plus Mesp1, and Myocd) was found to be more efficient at reprogramming human DFs, and CFs over GMTEM-based approach in generating iCMs that spontaneously contract and show action potential^88^.

Besides viral delivery of cardiac-specific transcription factors for direct cardiac reprogramming, Fu et al. reported small molecule-based reprogramming of mouse fibroblast. Using a combination of six small molecules CRFVPT (C: CHIR99021, R: RepSox, F: Forskolin, V: VPA, P: Parnate, T: TTNPB), the authors were able to reprogram mouse embryonic fibroblasts and tail tip fibroblasts into beating iCMs^89^. Chemically reprogrammed iCMs express typical CM markers (for example, Mef2c, cTnT, Gata4, α-MHC and α-actinin) and show CM electrophysiology. Unlike GMT-based reprogramming, fibroblast cells do go through a cardiac precursor stage when forming CMs^89^.

For chemical reprogramming of human somatic cells into CiCMs (chemically induced CMs), a combination of nine small molecule is required, referred as 9C (CHIR99021, A83-01, BIX01294, AS8351, SC1, Y27632, OAC2, SU16F and JNJ10198409 (JNJ))^90^. Compared with transcription factor-based GMTMM reprogramming, 9C-based chemical reprogramming yields higher iCMs resulting in 97% of the CiCMs spontaneously beating^90^. Moreover, transplantation of 9C-treated human foreskin fibroblasts into infarcted mouse hearts generated iCMs in vivo, which suggests targeted delivery of 9C factors into CF could also yield iCMs in treating MI^90^.

While inspiring, in vivo reprogramming of adult somatic cells remains one of the greatest challenges in regenerative biology. There are intrinsic limitations to in vivo somatic cell reprogramming, and despite tremendous advancements in engineering nonintegrating viruses, such as adeno-associated virus (AAV) and sendai virus, targeted delivery of a reprogramming virus, including coding sequencing for several transcription factors simultaneously into CFs in vivo will require additional development (Fig. 2c, left). Specifically, both dose and induction duration will need to be tightly regulated for in vivo cardiac reprogramming to be therapeutically applicable and may need to be complemented with adjuvant CMs proliferation or replacement strategies.

### EVs for cardiac repair and regeneration

Accumulating evidence argues that many of the cardioprotective benefits from cell therapy (MSCs or hPSC-CMs based) in animal MI models can be attributed largely to the paracrine effects of the transplanted cells, implying a potential role of naturally secreted EVs. This suggests that cardiac treatments with cell-free paracrine factors could attain many of the therapeutic benefits of cell therapies, including the protection and/or proliferation of resident CMs without many complications inherent to cell-based transplantation biology (for example, graft rejection, autoantibody and cardiac arrythmia)^91^.

First underappreciated, EVs have since received exceptional attention from the scientific community. At present, EVs serve as excellent biomarkers for an array of human pathophysiology, and many studies have also demonstrated functional roles for EVs in treating/repairing damaged tissues^92^. Given that EVs can be isolated from most biological specimens (body fluids and cells/tissues), it is not surprising that EVs are also implicated in normal cardiac development as well as cardiomyopathy, including MI. For instance, during fetal development in mice, placental tissue-derived EVs promote CMs maturation (an organized sarcomeres) and assist in fetal heart development (for example, increased heart rate and epicardial cell outgrowth)^93^. However, in response to acute MI, there is release of a large pool of CM, CF and endothelial-cell-derived EVs which boosts inflammatory cytokines production in infiltrating monocytes, further aggravating localized inflammation of the myocardium^94^. The pathogenic role of EVs in MI have been extensively reviewed by others, yet under normal physiological states, many EVs carry cardioprotective cargos, and in preclinical mice models of MI, EVs emanating from variety of cellular sources have been shown to render cardioregenerative effects^95^ (please refer to refs. ^96–98^ for additional details on the pathogenic roles of EVs). In this section, we will focus on the therapeutic roles EVs in cardiac repair and regeneration (Fig. 3).

#### EVs from cardiac-associated cell types

##### CM-EVs

In the course of generating functional hPSC-derived CMs for cell therapy applications, left-over conditioned media is enriched with EVs. Thus, EVs can be isolated at various stages of stem cell potency, progenitor stages of differentiation or post CMs differentiation (Fig. 2b). Recently, multiple groups have reported the isolation of human and mice PSC-derived cardiomyocyte-derived EVs (CM-EVs) with antiapoptotic, antihypertrophic and proangiogenic properties^99,100^ (Fig. 3b). These studies are described below.

CM-EVs are enriched with miRNAs implicated in angiogenesis (miR-218-5p, miR-122-5p, miR-25-3p, miR-92 and miR-21), antifibrosis (miR-1, miR-24, miR-25-3p and miR-218-5p) and M2 macrophage polarization (miR-21, miR-92 and miR-320a)^101^. Proteomics analysis further revealed that CM-EVs are filled with proteins that promote angiogenesis, endothelial cell movement/maturations and those that inhibit cardiac apoptosis^101^. Collectively, these results highlight how CM-EVs could be a better alternative to CMs in cardiac regeneration and post-MI therapy. Clinical translation of CM-EVs requires timely isolation of large quantities of EVs, and immature CMs (day 16) produce more EVs compared with mature CMs (day 25) and are probably better suited for scaling up^101^.

Santoso et al. (2020) reported isolating hiPSC-CM-derived CM-EVs that carry 97% of the parental cell miRNA, suggesting comparative paracrine potential between CM-EVs and CMs themselves^102^. In vitro, these CM-EVs protect hypoxic hiPSC-CMs from apoptosis by upholding mitochondrial membrane potential^102^. Treating MI mice with CM-EVs yielded substantial improvement on systolic and LVEF functionality and dramatically enhanced myocardium viability compared with control mice. CM-EVs also decreased infarct size and reduced fibrosis and cell death^102^. Mechanistically, CM-EVs appear to suppress the mammalian target of rapamycin (mTOR) signaling pathway and enhance autophagy to repair the hypoxic myocardium^102^. Besides protecting hypoxic CMs, CM-EVs also enhance angiogenic potential, migration and proliferation of endothelial cells, which suggest that CM-EVs also can render non-cell-autonomous cardioprotection^103^. Later, Gao et al. (2020) and Tominaga et al. (2024) also confirmed the cardioprotective and reparative functions of CM-EVs^101,104^. CM-EVs tend to improve gross heart functionality by suppressing CM death, promoting angiogenesis and by reducing localized inflammation via polarizing M1 macrophages to M2-like state^101^. hiPSC-CMs derived CM-EVs are not only superior to endothelial-cell-derived EVs and smooth-muscle-cell-derived EVs in terms of antiapoptotic, angiogenic and heart functionality, but CM-EVs also did not instigate cardiac arrythmias—a recurring complication associated with CMs transplantation^104^. CM-EVs were also observed to be up taken by the heart resident CMs, endothelial cells, fibroblasts, and macrophages, indicating that observed functional improvements to the myocardium are mediated through multiple cell types^101^. Thus, the cardioreparative effects of CM-EVs themselves, in many aspects of MI pathophysiology, may prove to be superior to CMs transplantation. This suggests that CM-EVs recapitulate many of the paracrine effects typically attributed to the positive outcomes of CM transplantation^102,104^.

When compared with hiPSC-CMs from heathy individuals, hiPSC-CMs derived from patients with left ventricular hypertrophy (LVH) shows distinct RNA compositions and EV functionality^105^. Using in vitro tube formations assay with hiPSC-derived endothelial cells, the authors demonstrated the healthy CM-EVs are significantly better than CM-EV^LVH^ in forming endothelial tubes, and CM-EV^LVH^ treated endothelial cells show altered gene expression profile compared with healthy CM-EVs^105^. Analogously, unlike healthy CM-EVs derived from neonatal rat CMs, CM-EVs from angiotensin II-induced hypertrophic CMs (CM^H^) enhance the production of inflammatory interleukin (IL-6 and IL-8) by the macrophages in vitro^106^. CM^H^ and their EVs appears to be enriched with miR-155, a proinflammatory miRNA, which is primarily responsible for activating macrophage mediated inflammation^106^. Therefore, patient genetic predispositions and health status may be important considerations when using autologous CM-EVs or allogeneic CM-EVs in interventional clinical trials.

Using hiPSCs and hiPSC-derived CMs, Liu et al. demonstrated that under hypoxic conditions, CM-EVs provide better protection to hiPSC-CMs when compared with pluripotent-stem-cell-derived EVs (Pluri-EVs)^99^. As anticipated, these CM-EVs are highly enriched with cardiac-specific miRNAs, such as miR-1 and miR-133a. Gene ontology analysis of CM-EV miRNA targets suggest a potential role for CM-EVs’ cargo miRNAs in mitigating cardiac muscle hypertrophy^99^. To promote longer retention in the infarcted rat myocardium and slow release of EV cargos in vivo, the authors encapsulated the CM-EVs within collagen patches maintaining EVs in the myocardium 24 h post implantation^99^. This significantly reduced the number of arrhythmic events, reduced cardiac dilation, increased ejection fraction and reduced the infarct size by diminishing apoptosis compared with PBS and hiPSC-derived EV patches on the MI rats^99^. In a recent report, González-King et al. further confirmed that under hypoxic condition, hiPSC-derived CM-EVs render superior antiapoptotic protection to hiPSC-derived CMs over hESC-derived EVs^100^, which suggest hypoxic preconditioning could be a beneficial addition to EV treatment regimes.

##### CDC-EVs

When endomyocardial biopsy tissues are expanded in vitro, they form multicellular cluster called ‘Cardiosphere’. Generally regarded as CDCs, cells derived from these clusters exhibit cardiogenic potential and express c-Kit and CD105^107^.

Both in vitro and in vivo experiments corroborate that angiogenic, cardioproliferative, and antiapoptotic functions of CDCs are recapitulated by CDC-derived EVs (CDC-EVs)^108^ (Fig. 3b). Administration of CDC-EVs in MI mice significantly improves LVEF, decreases infarct scar size and shows marked improvement in cardiac repair and functional recovery^108^. Treating CDCs with the GW4869, which prevents EV release, fails to recapitulate the therapeutic efficacy of the CDC-EVs, further indicating that EVs are responsible for the therapeutic potentials of CDCs. Mechanistically, cardioprotective miR-146a, which is enriched in CDC-EVs, may mediate this cardioprotection^108^. Gallet et al. also demonstrated similar cardioprotection benefits of human CDC-EVs using a swine MI model^109^. CDC-EVs render cardiac repair by decreasing resident CMs apoptosis and reducing inflammatory leukocyte infiltration at the infarct site^109^. These therapeutic benefits are more pronounced when EVs are administrated intramyocardially but not via intracoronary route as the former yield higher EV retention^109^. Thus, the administration route is critical for therapeutic efficacy of EVs since the route can influence heart-specific EVs enrichment^109^.

Reportedly, CDC-EVs provide better cardioprotection and repair in mice model of MI than MSC-derived EVs (MSC-EVs) owing to CDC-EVs’ superior ability to suppress inflammatory macrophage responses as compared with MSC-EVs^110^. Similar observation of CDC-EVs mediated suppression of inflammatory macrophage infiltration at the infarct site was also reported by de Couto et al. (2017) in rat and pig model of MI^111^. Mechanistically, the transfer of cargo miR-181b from CDC-EVs to the host macrophages and polarize macrophages from proinflammatory to anti-inflammatory state^111^. CDC-EVs cargos are also enriched (about 30%) with proteins associated with immune system and miRNAs targeting cardiac and immune pathways^112^.

A biodistribution study of human CDC-EVs demonstrates that a significant portion of intravenously (IV) injected CDC-EVs ends up in liver at 1 h post administration, with heart being the second highest for EV enrichment among organs^113^. Similar observations were found when CDC-EVs were injected 20 min after the ischemia/reperfusion (I/R) injury in mice; EVs were found within the left ventricles (about 30%) and predominately at the infarct site (about 60% of heart-enriched EVs)^113^. These observations suggest that there is some natural homing capacity in CDC-EVs when administered near the injured heart.

Similar to CMs, hypoxic preconditioning of CDCs (1% O_2_) significantly enhances angiogenic potential of CDC-EVs by upregulating proangiogenic miRNAs, including miR-126, miR-130a and miR-210 in the CDC-EVs^114^. Thus, minor-to-moderate hypoxic stress may elicit beneficial EV cargo loading, and this precondition strategy may become a routine procedure for therapeutic EV production.

##### CPC-EVs

Compared with more developmentally mature CM-EVs, cardiac progenitor cells (CPCs) have recently gained more attention as a stem/progenitor source of therapeutic EVs (Fig. 3a,b). The first report of CPC-derived EVs (CPC-EVs) was by Chen et al. in 2013. Sca-1^+^ CPCs overexpressing GATA4, were shown to release EVs (40–100 nm in size) in the surrounding media^115^. Using an in vitro oxidative stress-induced cardiotoxicity model, the authors showed CPC-EVs treatment markedly diminishes apoptosis in cultured rat heart myoblast cells (H9c2 cells)^115^. Furthermore, in a murine model of acute I/R, CPC-EVs treatment during reperfusion resulted in ~53% less apoptosis compared with PBS-treated mice. This may be mediated by GATA4-responsive miRNA cluster miR-144/451, which are known to ameliorate cardiac ischemic/reperfusion injury, and CPC-EVs are enriched with miRNA-451^115^. In addition to curbing apoptosis, CPC-EVs treatment also reduces the production of inflammatory immunoglobulin such as IgM, IgG1 and IgG4 by PBMCs^116^ and inhibits stimulated T cell proliferation, further supporting the anti-inflammatory benefits of CPC-EVs in post-MI therapy^117^.

Barile et al. (2014) first reported that CPC-EVs isolated from adult patients (undergoing heart valve surgery) exhibit strong antiapoptotic and angiogenic effect on cultured mouse CMs (HL-1) and human umbilical vein endothelial cells, respectively. These EVs were enriched with miR-210, miR-132, miR-146a-3p and miR-181^118^, of which their angiogenic and antiapoptotic function could be largely attributed specifically to miR-132 and miR-210. Moreover, in rat model of MI, human CPC-EVs administration significantly deceased infarct area and apoptosis with marked increase in LVEF^118^.

A recent study by Emmert et al. (2024) demonstrated that intracoronary but not intramyocardial administration of clinical grade CPC-EVs (CD63^+^, CD81^+^) isolated from adult donors improves LVEF parameters and decreases infarct size in acute MI porcine model^119^. Although this study suggests that mature/aged human CPC-EVs continue to maintain their cardioreparative/cardioprotective functionalities, CPCs isolation from aged donor itself is an invasive surgery. Moreover, it was previously established that compared with adult human CPCs, neonatal CPCs are remarkably better at in vitro proliferation (about 3.5-fold higher Ki67^+^ at P3 and increased c-Kit^+^ expression with subsequent passage) and demonstrate superior repair of infarcted myocardium (for example, LVEF, infarct area and fibrosis) in murine MI models^120^. Therefore, it reasonable to believe that CPC-EVs from neonatal or hPSC-derived CPCs would be superior to adult CPC-derived CPC-EVs for clinical applications, considering that routine isolation of adult CPCs may prove challenging as well.

Similar to hypoxic CM-EVs and CDC-EVs treatment in mice, hypoxic CPC-EVs show better angiogenic and antifibrotic effects over normoxic CPC-EVs. This is probably due to stress-induced enrichment of cargo miRNAs, such as miR-292, which mediate repair responses^121^. For example, CPC-EVs derived from c-Kit^+^ human CPCs (adult donor) under physoxia (5% O_2_) appears to show better angiogenic potential compared with normoxic CPC-EVs (21% O_2_). Physoxic condition also yields 1.6-fold more EVs from CPCs as compared with normoxic conditions^122^. Besides hypoxic pretreatment, it possible to potentiate CPCs further with treatments that augment EV release and/or enrich certain therapeutic cargos. For instance, ISX-9 (isoxazole)—a small molecule with demonstrated potential to enhance cardiac diffeentiation^123^—treated CPCs are enriched with miR-520/-373 family members, including miR-371, miR-302, miR-372, miR-373 and miR-520, as well as miR-512, miR-548 and miR-367 compared with control-treated CPCs. These miR-371 enriched CPC-EVs^ISX-9^ appears to harbor better antifibrotic potential than control CPC-EVs. In fact, CPC-EV^ISX-9^ showed better cardiac proliferation, less fibrosis, improved LV function and LVEF with compared with control and hiPSC-derived Pluri-EVs^124^.

Using a chemotherapy-induced cardiomyopathy mouse model, Desgres et al. (2023) demonstrated that hiPSC-CPC-derived CPC-EVs treatment improves LV functionality and enhances animal survival^125^. Considering many patients with cancer develop cardiomyopathy and experience heart failure, CPC-EVs could be a potential therapy to mitigate chemotherapy-induced cardiotoxicity as well. Collectively, the above CPC-EV studies have led to a very recent (2024), first-of-its-kind human clinical trial using CPC-EVs from hiPSC-derived CPCs on a patient with severely symptomatic with drug-refractory LV dysfunction secondary to nonischemic dilated cardiomyopathy^126^. Intravenous administration of CPC-EVs showed improved ECHO parameters coupled with increased LVEF over baseline after 6-months post transfusion with no sign of autoimmunity^126^. Though a single case study, these observations provide strong evidence that CPC-EV therapy represents a new paradigm in treating and reversing cardiomyopathies. Additional EV types, which may also effectively reduce fibrosis and improve CMs proliferation are described next.

#### Stem-EVs

##### MSC-EVs

Over the years, MSCs have been thoroughly investigated, not only due to their ability to differentiate into skeleton and cardiac lineages but also owing to their outstanding immunomodulatory paracrine effects^127^. Thus, MSC-EVs also provide anti-inflammatory and cardioprotective benefits. Relevant studies have been well reviewed by others; therefore, we review some of the more recent studies employing MSC-EVs for MI therapy here (Fig. 3b) (please review refs. ^128,129^ for additional details on MSC-EVs).

Lai et al. (2010) first reported that the cardioprotective effects of human MSCs (hESC-derived) are mediated through MSC-EVs using an ex vivo mouse model of cardiac I/R injury. The authors showed that purified MSC-EVs significantly decreased infarct sizes compared to conditioned media^18^. Xiao et al. further confirmed that the cardioprotective effects, primarily antiapoptotic and antiautophagic flux observed in mouse MI model following MSCs transplantation, is attributed to enrichment of miR-125b-5p in MSC-EVs; inhibiting EV biogenesis by GW4869 or administrating anti-miR-125b-5p abrogates cardioprotection. These observations strongly argue that MSC-EVs, rather than parental MSCs, render most cardioprotection benefits^130^. Arslan et al. (2013) further showed that a single dose of MSC-EVs (hESC-derived) before reperfusion decreases infarct size by approximately 20% (ref. ^131^). MSC-EVs administration appears to (1) reduce oxidative stress at the infarct site, (2) enhance cell survival through upregulation of PI3K/Akt signaling, (3) decrease phosphorylation of c-JNK and proapoptotic signaling and (4) reduce infiltration of proinflammatory leukocytes (neutrophil and macrophage) at the infarct^131^.

Xu and colleagues recently compared MSC-EVs isolated from human BMMSCs, adipose tissue‐derived MSCs (ADMSCs), and umbilical cord blood‐derived MSCs and evaluated their therapeutic efficacy using a rat MI model^132^. Compared with parent cells transplantation, all MSC-EVs types showed significantly elevated LVEF with ADMSC-EVs showing the highest LVEF recovery of all MSC-EVs types. ADMSC-EVs treatment also showed superior antiapoptotic activity and higher microvascular density over other MSC-EV types, which suggest that tissue-specific-origin of MSCs play some role influencing the therapeutic efficacy of different MSCs and their EVs^132^.

While characterizing MSC-EVs cargo, Peng et al. observed that mouse BMMSC-EVs were enriched with cardioprotective miRNAs, notably miR-21a-5p, miR-22-3p, miR-25-3p, miR-214-3p, miR-320-3p, which suggest that exosomal miRNAs play pivotal roles in MSC-EVs mediated cardioprotection^133^. In MI models, significant reduction in localized inflammation was marked by suppression of proinflammatory IL-1β, IL-6 and TNF-α expression, arguably contributing to reparative effects^134^. In addition to apoptosis, in a mouse model of hypoxia/reoxygenation injury, human BMMSCs-MSCs treatment significantly suppressed NLRP3/caspase-1-induced pyroptosis of primary mouse CMs by inhibiting NLRP3 expression via exosomal miR-320b. Considering elevated pyroptosis also manifests in myocardial I/R injury^135^, the transfer of cardioprotective exosomal miRNAs from MSC-EVs to receipt cells could be one of the primary mechanisms underlying MSC-EV-mediated cardioprotection^135^.

Similar to cardiac-origin cells, preconditioning MSCs with hypoxia or proinflammatory cytokines enhances the cardioprotective capacity of MSC-EVs. Administrating MI mice with MSC-EVs isolated from mouse BMMSCs treated with TNF-α shows reduced infarct size as well as elevated polarization of proinflammatory-M1 macrophages to anti-inflammatory-M2 macrophage over control BMMSC-EVs^136^. Similar to other studies, Wu et al. (2024) recently demonstrated that hypoxic BMMSCs generated EVs treatments also leads to smaller infarct areas compared with control BMMSC-EVs in a mouse model of ischemic stroke^137^. Similar observation was also found in a murine MI model where hypoxic mBMSC-EVs provides better cardioprotection than normoxic mBMSC-EVs^138^. Not only did hypoxic mBMSC-EVs treated mice exhibited better survival (compared with sham mice), but they also exhibited smaller infarct size, with improved LVEF and higher capillary density. These studies suggest improved neovascularization occurs with hypoxic over normoxic mBMSC-EV treatment^138^. Mechanistically, hypoxic treatment tends to elevate cardioprotective miR-210 expression in BMMSC-EVs; the internalization of miR-210 enriched MSC-EVs by resident CMs probably enables CMs to proliferate more, as evidenced by increased number of Sca-1^+^ CPCs in the infarct area^138^.

##### HSPC-derived EVs

In 2011, Sahoo et al. first reported that human CD34^+^ HSPC-derived EVs have angiogenic properties, promote the formation of vessel-like endothelial structures in vitro and improved vessel growth in corneal angiogenesis assays^139^. Using an ischemic hindlimb mouse model, they further showed that the proangiogenic property of human CD34^+^ EVs are largely attributed to the EV cargo carrying proangiogenic miR-126-3p^140^. Additional studies are needed to rank the cardioprotection capabilities of HSPCs-EVs to that of MSC-EVs, Pluri-EVs and cardiac lineage EVs to determine their effectiveness as post-MI therapies (Fig. 3b)

##### Pluri-EVs

Since the derivation of the first hESCs line in the year 1998^141^ and the seminal generation of iPSCs^142,143^, the regenerative medicine field has been flourishing with studies highlighting the remarkable potentials of hPSC-derived cells in repairing damaged tissues and, potentially, in engineering functional organ components. hPSC-derived CMs and their virtually unlimited scalability have gained substantial attention to replenish lost or damaged CMs following MI as discussed previously^144^ (Figs. 2b and 3a). Now, with recent advancement and exploration of EVs from different stem cells sources for cardiac regeneration, hPSC-derived EVs (Pluri-EVs) are also drawing significant attention (Fig. 3a). Pluri-EVs cargo contains many pluripotency, developmental, and immune regulators, including pluripotency-associated transcripts *OCT4*, *NANOG*, *SOX2,* cardiac-specific *GATA4* and a range of regulatory miRNAs^145^. Thus, Pluri-EVs themselves may also render cardioprotective benefits (Fig. 3b).

hiPSC-derived Pluri-EVs were first isolated by Bobis-Wozowicz et al. in 2015, when the authors uncovered that Pluri-EVs were enriched in pluripotency factors, miRNAs and other bioactive cargos. EVs were internalized by recipient heart-derived mesenchymal stromal cells (cMSCs)^146^, and pluri-EV treatment not only influenced global gene expression pattern of cMSCs but also promoted cMSCs proliferation under hypoxic conditions^146^.

Pluri-EVs also have been shown to influence immune response post-acute ischemic stroke^147^. In a recent study, Xia et al. demonstrated that the administration of hESC-derived Pluri-EVs in a mouse model of acute ischemic stroke significantly ameliorate neuronal death and diminishes brain infarction by suppressing infiltration of peripheral leukocytes and neuroinflammation. This was largely due to marked increase in T_reg_ (CD4^+^CD25^+^FOXP3^+^) cells^147^. The systematic uptake of TGF-β, Smad2 and Smad4 enriched in Pluri-EVs by the resident T_reg_ activated TGF-β/Smad pathway and Foxp3 expression and promoted T_reg_ expansion^147^. Based on these observations, we can postulate that similar T_reg_-mediated cardiac protection could be achieved with Pluri-EVs administered post-MI.

Using mouse CF-derived-iPSCs and a murine model of cardiac ischemia, Wang et al. demonstrated that administrating miPSC-derived Pluri-EVs immediately after the ischemia incident also reduces CM death, and this cardioprotective function appears to be superior to CF-EVs^148^. Cardioprotection mediated by miPSC-derived Pluri-EVs was attributed to the enrichment of miR-21 and miR−210 in the Pluri-EVs compared with CF-EVs; these miRNAs were also cardioprotective in myocardial ischemia^149,150^. Similarly, Khan et al. showed mESC-derived Pluri-EV treatments can significantly improve LVEF and decrease infarct size in a mouse MI model^151^. mESCs Pluri-EVs substantially enhanced vascularization in MI mice, increased CMs proliferation up to approximately sixfold and decreased CMs apoptosis by ~30% compared with control mice. Moreover, Pluri-EV treated mice showed a significantly increased number of c-Kit^+^ and GATA4^+^ CPCs with an overall increase in CPCs proliferation within the heart. Thus, Pluri-EVs may function in both ischemic protections and in cardiac regeneration^151^ (Fig. 3).

Similar to MSC-EVs and CM-EVs, Pluri-EVs appear to be better at cardioprotection than parental PSCs^152^. Mouse Pluri-EV treated mice exhibit improved LV function with less myocardial hypertrophy compared with mock or miPSCs treated mice^152^. Unlike miPSCs, which generate teratomas in immunodeficient mice following intramyocardial injection, mouse Pluri-EVs provide cardioprotective/cardioproliferative miRNAs (miR-17–92, miR-294) to the infarcted myocardium without causing cancer^152^.

Recently González-King et al. compared cardioprotective benefits among hESC-derived Pluri-EVs, CM-EVs, CPC-EVs and BMMSC-EVs and demonstrated that Pluri-EVs exhibit superior angiogenic, antifibrotic and cardio-repair potential over CM-EVs, CPC-EVs and BMMSC-EVs^100^. Compared with CM-EVs, Pluri-EV treatment reduced mature collagen deposition and alpha-smooth muscle actin expression (α-SMA) in a in vitro model of cardiac fibrosis, using human primary ventricular CFs (VCFs)^100^. Direct intramyocardial injection of Pluri-EVs also increased LVEF and decreased infarct area compared with CPC-EVs or BMMSC-EVs treatment^100^. We have also observed (manuscript in preparation) that under hyperglycemic conditions, Pluri-EVs provide superior cardioprotection to hiPSC-CMs in vitro, compared with CM-EVs or MSC-EVs. Collectively, these reports imply that human Pluri-EVs may represent strongest candidate for treating patients with MI among EVs derived from CM, CPC and BMMSC.

Analogous to hypoxic treatment of MSCs, CPCs and CMs, culturing hPSCs under hypoxic condition (5% O_2_) generates hypoxic Pluri-EVs^(hypo)^ that have superior antifibrotic property, owing to the enrichment of miR‑302b‑3p in EV cargo^153^. Hypoxia does not alter expression of core pluripotency factors in the parental hiPSCs^153^. Bobis‑Wozowicz et al. (2024) further showed that hypoxic Pluri-EVs^(hypo)^ also provide greater CMs protection; Pluri-EVs^(hypo)^-treated CMs exhibit markedly decreased level of oxidative stress and inflammation in addition to elevated prosurvival signaling cascade in an in vitro oxygen‑glucose deprivation/reoxygenation model of CM oxidative damage^154^. These observations corroborated the beneficial effect of hypoxic treatment by Nakada et al.; they demonstrated that exposing mice to hypoxic condition (7% O_2_) significantly decreases reactive oxygen species buildup and oxidative DNA damage in CMs, along with marked CMs proliferation in vivo as compared with normoxic conditions^155^. Furthermore, following MI, hypoxic treatment alone reduces fibrotic scarring, increasing CMs and vascular cell proliferation and improves survival of the mice over normoxic treatment^155^. It would be of great interest to investigate whether hypoxic Pluri-EVs provide superior recovery/repair following MI in vivo as well.

## Current limitations of EV therapy

A growing body of evidence indicates that compared to cell therapy, EV therapy provides comparable and, in many cases, superior cardioprotection in post-MI recovery/repair^18,102^. Despite inspiring preclinical data and promising early outcomes in recent clinical trials, stem-EV-based therapies are still in their infancy, necessitating further advancement to reach clinical translation (Fig. 4b).

### Adaption to the suspension culture

Originally grown on a range of adhesion matrices^156^, many stem cell cultures have been propagated under scalable, often suspension-based Good Manufacturing Practices (GMP) systems^66^, which should streamline the adoption of cell-secreted EVs purified directly from these formerly discarded media^157–159^. Highly automated and physiologically tunable suspension culture bioreactors may prove useful for some cell types and EV production^160^; however, for most EVs, it has yet to be determined whether switching from adherent/monolayer to suspension cultures/bioreactors may significantly affect EV cargo content and/or overall EV yields. Since minute alteration in the culture milieu (for example, aeration, CO_2_ level, nutrient level and so on) could affect EV cargo composition, the entire EV manufacturing process will need to stringently monitored and regulated to produce GMP-grade Stem-EVs for routine clinical usage^161^. Off the shelf products are desired, and several solutions to long-term EV storage have been recently reviewed^162,163^.

### Large- or industrial-scale EV purification

EVs are routinely isolated from conditioned media using ranges of purification methods, such as ultracentrifugation, commercial isolation kits, size exclusion chromatography and tangential flow filtration (TFF)^164^. Though methodological differences influence EV quality and yield, clinical applicability of EVs demands the highest purity and yield, devoid of protein or other cellular contaminants to fulfill regulatory requirements. From our own experience, TFF is compatible with large scale EVs isolation from suspension culture (2–10L) which yields up to 10^11^ particles per milliliter; however, cellular protein contaminants do persist in the TFF-derived EV elutes. Therefore, improved/new purification method which provide GMP-grade EVs without significant loss of particles number and quality should be given high precedence.

### Reporting of cell- or tissue-specific EV markers

MISEV2023 guidelines require that EV characterization should be accompanied by morphological assessment of EVs using TEM and validation of standardized EV markers CD9, CD63 and CD81^32^. However, given the heterogenous nature of EVs, EV sources, conditioned media and cell culture vessel (adherent versus suspension), tissue-specific EV markers, particularly for Stem-EVs, should be characterized and reported as part of FDA requirement before Stem-EVs clinical implications (Fig. 4). For reference, Chen et al. have recently established that PODXL plus SSEA4 are reliable cell-surface markers for human Pluri-EVs^165^. Other technical advances, including EV-specific flow cytometry (Beckman CytoFLEX nano machine) or single-molecule localization microscopy-based EV characterization (Bruker Vutara VXL machine, ONI Nanoimager), may streamline EV characterization at lower sample inputs and may facilitate timely FDA approval for EV-based therapies for cardiac repair.

### Dosage and administration route of EVs

Another area that requires significant attention is the dosing of EVs (the tentative number of EV particles/injection or number of particles per kilogram of patient weight, required to exert therapeutic benefits) and the optimal route of EV administration. In preclinical animal models, EVs are routinely administrated either through tail vein injection or direct intramyocardial inject to the infarcted heart, typically at a rate of 10^9^–10^10^ EVs per dose with multiple dosing regimen. Similar dosing is unlikely compatible for human patients. For instance, recently, Menasché and colleagues have reported that subjects with a non-MI cardiopathy in clinical trial were treated with 20–40 × 10^9^ particles per kilogram of GMP-grade CPC-EVs purified by TFF^126^, requiring significantly more EVs. Thus, in vitro experiments should test multiple isogenic cell lines to determine high yielding EVs producer line that can be scaled up for human clinical translation of EV therapies. Although our aforementioned discussion signifies how intramyocardial EV administration yield superior recovery over IV or other delivery route, for end stage patients with heart failure or patients with age-associated complications, intramyocardial injections, especially, are invasive with potentially fatal complications. As a potential alternative, in a recent clinical trial, one patient received CPC-EVs administrated intravenously^126^. Though intravenous injections are routinely used for delivering a wide spectrum of drugs, this route may not deliver EVs directly to the MI site, and the majority of intravenously introduced EVs may end up in the liver^113^. Thus, targeted EV delivery has become a promising area of development; engineering EVs with cardiac specific signal peptides may improve delivery of EVs to cardiac cells targets via circulation (Fig. 4). Until then, epicardial administration of Stem-EVs via the pericardial sac could be an alternative route for localized EV targeting to the heart with minimal postoperative complications^166^.

## Future directions for EV therapy

EV therapy holds unprecedented potentials for cardiac repair and regeneration with new developments emerging every day. Major areas of active development in the EV field, which may transition EV therapy closer to clinical applications (Fig. 4c), are discussed below.

### Engineering EVs to enhance therapeutic efficiency

Most types of EVs investigated for MI therapy carry native therapeutic cargos that largely reflect the molecular profile of their parental cell of origin (Fig. 4a). Nevertheless, recombinant EVs (rEVs) are being engineered by overexpressing biomolecules (for example, factors, miRNAs or lncRNAs) in the parental cells, to further elevate the therapeutic functionality of EVs. For instance, compared with naive EVs, rEVs from miRNA-181a overexpressing hUCMSCs show markedly reduced level of TNF-α and IL-6 expression (proinflammatory) and elevated levels of IL-10 (anti-inflammatory) in stimulated hPBMCs^167^. The percentage of T_reg_ cells is also significantly higher in rEV-treated hPBMC population compared with naive EVs^167^. Furthermore, MI mice receiving intramyocardial injection of miRNA-181a-enriched rEVs demonstrate elevated LVEF and smaller infarct size compared with those receiving naive EVs^167^. Initially identified as a negative regulator of *Ngal* (lipocalin-2) in the aldosterone-mineralocorticoid receptor (Aldo-MR) pathway^168^, miRNA-181a has been known to play a cardioprotective role. Knockout mice lacking miRNA-181a exhibit exacerbated heart function (LVEF) and fibrosis post-MI compared with control mice^168^, whereas mice overexpressing miRNA-181a show significantly reduced apoptosis, smaller infarct size and relatively higher LVEF, probably through suppression of proapoptotic programmed cell death protein 4 (PDCD4)^169^. Analogously, compared with naive EVs, rEVs from rat BMMSCs overexpressing miR-183-5p showed superior cardioprotection both in vitro and in MI rats partly due to their ability to suppresses *FOXO1* and alleviate apoptosis and oxidative stress^170^. Considering MI rats overexpressing miR-183-5p exhibit cardioprotective benefits that are comparable to those of miR-183-5p-enriched EV treatment^170,171^, miR-183-5p has emerged as a promising recombinant cargo for EV-mediated MI therapy.

In addition to miRNAs, treating MI rats with growth differentiation factor 15 (GDF15)-enriched rEVs also markedly reduces infarct size and improves LVEF compared with PBS or naive EV treated controls, probably through mitigating post-MI inflammation, cardiac apoptosis and the promotion of angiogenesis^172^. Comparable cardioprotective enhancement was observed in MI rats treated with GDF15-enriched macrophage EVs, relative to naive ones^173^. Besides overexpressing GDF15 in the parental cells, preischemic conditioning of MI-rats with GDF15 also tend to reduce infarct size in vivo and elevate LVEF, as assessed ex vivo^174^. Furthermore, pretreating hMSCs with GDF15 not only enhances their antiapoptotic, antioxidative stress and other therapeutic functions in vitro but also improves their post-transplantation survival in MI mice, resulting in smaller infarct size and elevated LVEF relate to control MSCs^175^. Although elevated circulating GDF15 level is typically associated with poor prognosis in patients with coronary heart diseases including MI^176,177^, GDF15-enriched rEVs appear to be cardioprotective, warranting further investigation.

Apart from enriching miRNA/factors in EVs to enhance therapeutic efficacy, a recent report by Park et al., suggests that depleting deleterious cargos from the EVs could also enhance their therapeutic potential^178^. The authors demonstrated that EVs derived human cardiac c-Kit^+^ cell (CPCs) with knockdown of miR-192-5p and miR-432-5p exhibit enhanced antifibrotic, anti-inflammatory and improved angiogenesis, both in vitro and in a rat model of ischemia–reperfusion compared with naive CPC-EVs^178^. This observation strongly argues for detailed characterization of EV cargo derived from different cellular origin and their comparative analysis before their clinical implications.

### Priming EVs to enhance therapeutic functions

Priming or preconditioning the host cells with external stimuli or growth factors/cytokines to enrich EVs with distinct cargos or receptors has garnered significant attention lately. In earlier sections, we discussed how treating stem cells or other cardiac-associated cells with hypoxia generates EVs with improved functionality and/or cargo composition, enhancing their competence in repairing or regenerating infarcted myocardium. In a recent study, Li and colleagues have used BMMSCs derived from a nonhuman primate (monkey) and have further substantiated that treating MI mice with hypoxic (0.5% O_2_) BMMSC-EVs improves LVEF, reduces infarct size and enhances angiogenesis relative to normoxic EV treatment^179^. Mechanistically, hypoxia appears to enrich miR-486-5p in the EV cargo. Upon treatment, miR-486-5p-enriched hypoxic EVs are internalized by CFs, where miR-486-5p downregulates matrix metalloproteinase 19 (*Mmp19*) to maintain VEGFA levels, thereby enhancing angiogenesis in the infarcted heart^179^. Overexpressing miR-486-5p in normoxic BMMSCs also enhances their cardiac repair and angiogenesis potential^179^, indicating that miR-486-5p is a promising candidate for EV engineering.

In addition to hypoxia, proinflammatory stimuli represent another promising priming strategy for generating superior EVs for MI therapy. Recent studies by Shen et al. have demonstrated that interferon γ (IFNγ) treated mouse adipose-derived MSCs generate inflammation-primed EVs (iEVs) that exhibit enhanced anti-inflammatory and scar healing properties compared with naive EVs, evaluated in a mouse model of achilles tendon injury and repair model^180,181^. The level of NF‐κB activity and the expression of proinflammatory genes *Ifng* and *Il1b* were significantly lower in iEVs treated tendons relative to naive EV treatment, with iEVs enhancing anti-inflammatory genes *Arg1*, *Il13* and *Il1rn* expression, indicating elevated M1-to-M2 polarization of the resident macrophages^173^. IFNγ priming does not affect EV yield^180,181^ yet enriches iEVs with miR-147^173^, a negative regulator of murine macrophage inflammatory responses^182^. Similarly, iEVs derived from hBM-MSCs loaded onto biphasic calcium phosphate biomaterial, primed with human IL1β and TNF-α, exhibit elevated M1-to-M2 macrophage polarization^183^. Hackel and colleages have reported that iEVs derived from human nasal mucosa MSCs primed with IFN-g, TNF-α and IL-1b exhibit markedly decreased prolieration of CD3^+^ T cells and significantly elevated induction of T_reg_ (CD4^+^ CD127dim CD25^+^ FOXP3^+^)^184^. The elevated level of PD-L1 and PD-L2 (programmed death ligand) on immuno-primed MSCs (probably in the iEVs membrane as well) appear to be reposible for T_reg_ activation by iEVs^184,185^. Similarly, iEVs from TNF-α, IFN-γ and IL-1β-primed UCMSCs shows increased FOXP3 expression on activated PBMCs relative to naive EVs, suggesting enhanced anti-inflammatory potential^186^. Given the profound role of proinflammatory cascade in MI-pathogenecity, generating stem-cell-derived iEVs with enhanced M1-to-M2 macrophage polarization and T_reg_ induction potential could be a promising addition to EV-based therapeutic toolkit.

It should be noted that compared with naive EVs, UCMSC-derived iEVs has been reported to retain elevated level of let-7f-1-3p, miR-139-5p, mir-140-5p, miR-193a-5p and miR-214-5p which are implicated in the negative regulation of cell proliferation^186^. Moreover, cargo proteomics analysis of iEVs from TNF-α, IFN-γ and IL-1β-primed UCMSCs exhibit significant enrichment of proinflammatory proteins such INF-γ, CS F-1, MCP2, MCP4 and CCL3, relative to naive UCMSC-EVs^187^. These observations highlight the significance of defining and fine-tuning priming parameters (concentration of the drug/treatment, duration and appropriate host cells) and systematically comparing EV cargo or proteomes for possible alternations.

### Cardiac-targeted delivery and enrichment of therapeutic EVs

EV biodistribution studies in murine models corroborate that a significant portion of the administrated EVs (>50%) end up in liver, spleen, lungs and gastrointestinal tract^188^, regardless of the influences of route of administration or parental cell of origin^189^. A promising strategy to circumvent such off-target EV delivery and enhance the enrichment of therapeutic EVs in the heart involves incorporating cardiac targeting peptide (CTP) into EVs^190,191^. CTPs are small, naturally occurring or synthetic, comprising ~6–30 amino acids and exhibit innate cardiac homing capacity^180^. One of the most widely utilized CTP, APWHLSSQYSRT, was identified in 2010 through a phage peptide display library screening in mice. It demonstrated a strong in vitro transduction efficiency in H9c2 cardiomyoblast cells^190^ and a recent follow up study reported no toxicity, both in vitro and in vivo, following CTP administration^192^. A series of study by Kim et al. subsequently demonstrated that HEK293 cells overexpressing CTP fused to LAMP2b (lysosome-associated membrane protein 2b), an EV-associated transmembrane protein, generate CTP-EVs with enhanced cardiac-specific cellular uptake, both in vitro and in vivo^193,194^. Following tail vein injection, CTP-EVs exhibit ~15% higher enrichment in mice heart compared with control EVs^170^. As anticipated, CTP overexpression does not affect EV morphology or zeta potential of the EVs and remains nontoxics to receipt rat CMs^194^. Compared with naive EVs, CTP-EVs shows an approximately twofold higher accumulation in rat’s heart (tail vein injection) despite liver specific enrichment which further underscores the significance of CTP in circumventing off-target and cardiac-specific EV enrichment for MI therapy^194^. Using a different CTP sequence (WLSEAGPVVTVRALRGTGSW)^195^, Mentkowski et al. also demonstrated significantly elevated enrichment of CDC-derived CTP-EVs in the mice heart following intramyocardial injection^196^. When administrated near the heart, CTP-EVs achieves over fourfold higher cardiac enrichment compared with control EVs, suggesting that CTPs not only augment EV homing while in circulation but also enhance their heart-specific internalization^196^.

In addition to general-purpose CTPs discussed above, Kanki et al. have identified an ischemic myocardium-targeting peptide, CSTSMLKAC (IMTP), which appears to facilitate highly selective homing to ischemic heart tissues^197^, making IMTP a compelling candidate for EV-based MI therapy. Using IMTP-EVs derived from mouse BMMSCs, Shen and colleagues recently demonstrated that compared with control EVs, IMTP-EVs exhibit ~1.8–2-fold higher enrichment in infarcted mouse hearts^198,199^. It would be of great interest to compare CTP-EVs and IMTP-EVs, derived from same host cells, side-by-side to determine which one would be a stronger candidate for EV-based MI therapy.

While novel CTPs with higher specificity and pharmacodynamics are being actively developed and pursued for cardiac-specific EV enrichment^200^, the cargo profile of CTP-EV or IMTP-EV, particularly those derived from stem cell lines, should be thoroughly characterized and systematically compared to naive EVs before their clinical applications.

### Enhancing EV internalization and prolonged circulatory retention

Intracellular EV uptake is an inherently inefficient process—only ~1% EVs are spontaneously internalized within the first hour^201^; however, a dose- and time-dependent increase in EV internalization has been reported^202,203^. A considerable portion of internalized EVs transition from early endosomes to late endosome, eventually being degraded in the lysosome whereas only a small fraction of early endosomal EVs are released into the cytoplasm, enabling their cargos to be functional^204,205^. These rate-limiting steps not only suppress EV’s effectiveness in vivo but also necessitate higher EV doses to exert measurable therapeutic effects. To this end, enhancing intracellular EV uptake, endosomal escape and limiting cellular clearance can elevate clinical applicability of EV therapy for MI.

Cell penetrating peptides (CPPs) are a group of small peptides (<30 amino acids), which not only can translocate inside the host cells, CPP-conjugated complex/particles readily internalize via different endocytosis pathway^206^. Extensively leveraged in drug delivery, gene/RNA therapy and diagnostics, some recent studies underscore tremendous potential of CPP-modified EVs for improving cellular EV uptake. To this end, Nakase and colleagues have demonstrated that EVs modified with arginine-rich CPP such as Stearyl-r8 ((CH_3_(CH_2_)_16_-CO-NH-_D_(Arg)_8_-amide))^207^ or EMCS-R8 ((CH_3_-CO-NH-Cys(EMCS)-Gly-(Arg)_8_-amide))^208^ result in significantly greater cellular uptake compared with naive EVs, primarily through active micropinocytosis. CPP-tagged EVs exhibit limited to no cytotoxicity with Stearyl-r8-EVs demonstrating approximately 33-fold higher cellular uptake^207^. Recent reports by Hirase et al. and Zhang et al. further substantiate that CPP-modified EVs exhibit superior cellular uptake compared with their naive counterparts with negligible to no cytotoxicity across various EV and host cell types^209,210^.

With regards to extending circulation time, Kooijmans and colleagues enriched polyethylene glycol (PEG) conjugated nanobodies into EVs using postinsertion method, which resulted in extended circulation (about 60 min) post injection, compared with unmodified EVs which were undetectable after just 10 min^211^. The incorporation of PEG in the EV membrane does not affect EV size and morphology, and the postinsertional PEG enrichment is adaptable to EVs from various cell types^211^. Implementing a similar postinsertion method, Patras et al. generated PEGylated EVs passively loaded with doxorubicin (DOX)^212^. When compared with long-circulating liposomes loaded with DOX and free drug (DOX), PEG-EV-DOX demonstrated enhanced cellular uptake and smaller tumor volume in a murine melanoma model^212^, which suggest that PEG-EVs loaded with therapeutic drugs (for example, curcumin) could be effectively adapted for MI therapy.

### Drug-loaded EVs for enhanced therapeutic functionality

The majority of pharmacological agents currently approved for acute MI treatment are small molecules targeting three primary mechanisms: (1) thrombosis and blood clot prevention (antithrombotic/antiplatelets, for example, P2Y12 receptor antagonist, PAR-1 inhibitor), (2) cardiovascular remodeling and hemodynamic control (neurohormonal modulators, for example, angiotensin II/aldosterone receptor blockers) and (3) metabolic and lipid regulation (metabolic protectors, for example, PCSK9 inhibitors, SGLT2 inhibitors)^213^. Although these drugs have dramatically improved patients’ quality of life post MI, the development of novel drug targets and antagonists remains crucial for addressing two major consequences of ischemic MI: CM apoptosis and local inflammation. In recent years, curcumin, a natural polyphenolic compound mostly found in the rhizome of turmeric plant, has drawn interest owing to its pleotropic therapeutic benefits ranging from anti-inflammatory to anti-apoptosis and across a range of age-associated metabolic conditions, in particular, for CVD^214^. In addition to the abundance of in vitro data supporting curcumin’s cardiac protective role, supplementing MI mice/rats with curcumin significantly reduces infarct size and fibrosis and elevates LVEF^215,216^, making curcumin an promising supplementation for MI therapy. Unfortunately, the limited bioavailability of curcumin restricts it clinical applicability^217^, and targeted cardiac delivery of curcumin via EVs could be a great solution to oral or intravenous administration. To this end, Sun et al. first demonstrated that the encapsulation of curcumin in EVs significantly enhances curcumin’s stability and systemic bioavailability—higher plasma concentration over free curcumin^218^. Kang and colleagues have further demonstrated that the encapsulation of curcumin in CTP-tagged EVs enhances its stability, and curcumin loaded CTP-EVs result in smaller infarct size and elevated LVEF in MI mice relative to CTP-EVs alone^219^. Moreover, codelivering curcumin and miR-144-3p into the infarcted heart via CTP-EVs results in enhanced post-MI recovery compared with CTP-EVs carrying curcumin alone^219^. Collectively, these observations make a compelling case for leveraging engineered EVs enriched with recombinant therapeutic cargo and curcumin as a promising combinational therapy for MI treatment.

## Closing remarks

Therapeutic EVs, in particular Stem-EVs, hold immense potentials for cardiac repair and regeneration in post-MI therapy. Inherent cardio-protective functions of most Stem-EVs (Fig. 3) imply many EVs can function as novel stand-alone biologics. Alternatively, EVs can be synergistically administrated during or following cell transplants to improve graft health and viability by preventing cell death, alleviating proinflammatory responses and augmenting electrophysiology. Yet, many circulating EVs promote aging and chronic illness^220–222^. Thus, cardiac repairing/regenerating EV treatments may also need to be combined with pathological EVs removal or suppression strategies^223,98^. Given the field is currently evolving, EV therapy still needs to overcome many existing hurdles before clinical adoption. There remain technological and logistic challenges, such as high-grade purification, cell-of-origin marker reporting, complete characterization of EV cargos and long-term storage management, and with FDA regulations specifying current GMP-grade EVs manufacturing, these challenges are actively being solved (Fig. 4b). Besides native Stem-EVs, engineered Stem-EVs carrying therapeutic cargos and/or drugs or modified membrane proteins for tissue-specific targeting are expected to flood the translational EV field (Fig. 4c). However, genetically modified EV-producer lines will probably have to strictly adhere to guidelines adopted for the genetically modified cell therapies, such as CAR T cell therapies^224^. In conclusion, we expect Stem-EVs from variety of stem cell sources will be the next generation of cell-free biologics for treating MI and other cardiovascular complications.
